# Knowledge of Cardiovascular Disease Risk Factors and Warning Signs Among Adults in the Jazan Region, Saudi Arabia: A Cross-Sectional Study

**DOI:** 10.3390/healthcare14132002

**Published:** 2026-07-06

**Authors:** Hossam Shaabi, Hassan Jaafari, Naif Gharwi, Raghad Bajawi, Raneem Zakri, Taif Hakami

**Affiliations:** Department of Surgery, College of Medicine, Jazan University, Jazan 14542, Saudi Arabia; hassanxjaafari@gmail.com (H.J.); fgghks@gmail.com (N.G.); raghad28@outlook.sa (R.B.); ranzakzakri@gmail.com (R.Z.); taifkhalid54@gmail.com (T.H.)

**Keywords:** cardiovascular disease, knowledge, risk factors, warning signs, Saudi Arabia, Jazan, cross-sectional study

## Abstract

**Highlights:**

**What are the main findings?**

- More than half of adults in the Jazan region (53.7%) had inadequate knowledge of cardiovascular disease (CVD) risk factors and warning signs, despite high general awareness of CVD (89.5%).
- Knowledge of warning signs (mean 2.96/10) was lower than knowledge of risk factors (mean 3.95/11), with chronic kidney disease and jaw/teeth pain the least recognized.

**What are the implications of the main findings?**

- University education (aOR = 2.44) and family history of chronic disease (aOR = 2.26) independently predicted adequate CVD knowledge, identifying populations that should be prioritized in regional health-education planning.
- The persistent awareness–knowledge gap supports a shift toward specific, actionable CVD education delivered through digital platforms and primary-care providers, rather than generic awareness messages.

**Abstract:**

**Background:** Cardiovascular diseases (CVDs) are the leading cause of death in Saudi Arabia, and public knowledge of risk factors and warning signs supports early detection and prevention. This study aimed to assess CVD knowledge and its demographic predictors among adults in the Jazan region. **Methods:** A cross-sectional study was conducted among 382 adults (≥18 years) between February and April 2025. A questionnaire adapted from prior validated instruments assessed CVD awareness, knowledge of 11 risk factors and 10 warning signs, perceptions, and practices. Total knowledge scores (0–21) were dichotomized as adequate (≥8) versus inadequate (<8). Mann–Whitney U and Kruskal–Wallis tests were used for bivariate analysis, followed by binary logistic regression. **Results:** Most participants (89.5%) had heard of CVD, yet 53.7% had inadequate knowledge, and only 9.9% demonstrated good knowledge (≥15). The median total knowledge score was 7 (IQR 2–11) out of 21, with warning-sign knowledge (2.96/10) lower than risk-factor knowledge (3.95/11). Overweight/obesity (52.6%), hypertension (51.3%), and smoking (49.5%) were the most recognized risk factors; chest pain (47.6%) and shortness of breath (46.1%) were the most recognized warning signs. University education (aOR = 2.44, 95% CI 1.23–4.85, *p* = 0.011) and family history of chronic disease (aOR = 2.26, 95% CI 1.32–3.85, *p* = 0.003) were the only independent predictors of adequate knowledge. **Conclusions:** More than half of the surveyed adults in the Jazan region had inadequate CVD knowledge despite high general awareness. These findings suggest that targeted education using digital platforms and primary care providers may help improve knowledge of risk factors and warning signs in the region.

## 1. Introduction

Cardiovascular diseases (CVDs) remain the leading cause of mortality worldwide, accounting for approximately 17.9 million deaths annually, which represents nearly 32% of all global deaths [1]. The burden of CVDs is disproportionately rising in low- and middle-income countries undergoing epidemiological and nutritional transitions, where modifiable risk factors such as hypertension, diabetes, obesity, smoking, and physical inactivity are increasingly prevalent [2,3]. Crucially, most CVD-related deaths are preventable through early identification and management of these modifiable risk factors.

In Saudi Arabia, CVDs constitute a major public health concern, accounting for approximately 45% of all deaths [4]. The country has experienced rapid urbanization and major lifestyle changes over recent decades, leading to alarming increases in obesity, diabetes, dyslipidemia, and sedentary behavior [5,6]. These trends are further compounded by the country’s young demographic profile, as CVD risk factors established during youth tend to persist and worsen with age [7]. Recognizing this challenge, Saudi Vision 2030 has prioritized reducing the burden of noncommunicable diseases and increasing life expectancy from 75 to 80 years through national prevention strategies [4].

Public knowledge of CVD risk factors and warning signs supports early detection and timely treatment-seeking behavior. Awareness of modifiable risk factors empowers individuals to adopt preventive health behaviors, including dietary modification, regular physical activity, and smoking cessation [4]. Internationally, studies from Jordan, Kuwait, Tanzania, Bangladesh, and sub-Saharan Africa have consistently reported limited public CVD knowledge, with education level and place of residence emerging as significant predictors of awareness [8,9,10,11].

Within Saudi Arabia, several regional studies have assessed CVD knowledge with varying results, generally reporting moderate-to-inadequate levels of awareness across different populations and settings [12,13,14,15,16,17,18]. In the Jazan region, existing research has focused on hypertension awareness in rural primary care settings [19], hypertension knowledge among hypertensive patients [20], chronic kidney disease awareness [21], and routine medical checkup knowledge [22]. However, no general-population survey has assessed overall CVD knowledge, including awareness of risk factors, warning signs, preventive behaviors, and emergency response, among the general adult population in the region. The present study addresses this gap with a region-specific assessment that applies a single validated instrument covering both CVD risk factors and warning signs, rather than either domain alone, thereby enabling direct comparison of the two knowledge components within the same population. Two features distinguish the Jazan region from areas where CVD knowledge has previously been surveyed: fat- and oil-rich dietary habits [23], and a higher proportion of rural and remote communities than the central and western regions. These features plausibly affect both CVD risk-factor exposure and access to public health messaging. Whether they translate into knowledge patterns that differ from those of Saudi populations in other regions has not been documented.

Therefore, this study aimed to assess the level of knowledge regarding CVD warning signs and risk factors among adults in the Jazan region, Saudi Arabia. Specifically, the study sought to determine the level of CVD awareness, identify demographic predictors of knowledge, and evaluate preventive behaviors and emergency response knowledge related to CVD. The findings are intended to provide an evidence base for designing targeted health education interventions to enhance early detection and reduce the burden of CVDs in the region.

## 2. Materials and Methods

### 2.1. Study Design and Setting

A cross-sectional observational study was conducted in the Jazan region, Saudi Arabia, located along the Red Sea coast adjacent to the Yemeni border and comprising both urban and rural communities. Data were collected between 24 February and 7 April 2025. The study is reported in accordance with the STROBE Statement for cross-sectional studies.

### 2.2. Study Population and Eligibility Criteria

The target population included adults aged 18 years or older residing in the Jazan region, Saudi Arabia. Individuals working in medical or paramedical fields and students enrolled in health-related programs were excluded to ensure that the assessment reflected general public knowledge without inflation from professional medical training.

### 2.3. Sample Size Determination

The minimum required sample size was calculated using the formula *n* = Z^2^ × P(1−P)/d^2^, where Z = 1.96 (95% confidence level), P = 0.30 (assumed prevalence of adequate CVD knowledge based on prior regional studies reporting suboptimal awareness [14]), and d = 0.05 (5% margin of error), yielding a minimum of 323 participants. A total of 382 valid responses were collected, exceeding the required minimum. This sample also satisfies the events-per-variable rule (EPV ≥ 10) for binary logistic regression with 12 predictor parameters [24].

### 2.4. Sampling Strategy and Data Collection

A convenience sampling approach was employed. A self-administered Arabic-language questionnaire was developed using Google Forms and distributed electronically via social media platforms, including Snapchat, Telegram, WhatsApp, and X (formerly Twitter). Submissions were limited to one response per Google account using the “limit to one response” setting, which required respondents to sign in and thereby reduced duplicate entries; restriction by IP address was not applied. Electronic informed consent was obtained on the first page of the questionnaire; participants who declined were directed to exit the survey. All items were marked as required; consequently, submitted forms contained no missing data, and incomplete forms could not be submitted and were therefore not captured. The number of individuals who opened the survey link was not recorded, so the response rate among those who viewed the survey could not be calculated. A pilot study was conducted with 30 participants to evaluate the clarity and readability of the questionnaire; no modifications were warranted, and pilot responses were not included in the main analysis.

### 2.5. Study Instrument

The questionnaire was developed based on a validated instrument originally used to assess CVD knowledge among young and middle-aged adults in rural Tanzania [25], which was subsequently translated, adapted, and validated in Arabic for the Syrian population by Swed et al. [26]. The same Arabic-language instrument was used in the present study, with only minor lexical adjustments for Saudi Arabian usage; content domains were preserved to maintain direct comparability with the parent studies. The adapted version was reviewed by two bilingual subject-matter experts (one cardiologist and one public health specialist) for cultural appropriateness and clarity prior to distribution. The review was qualitative; each item was assessed for relevance and comprehensibility, and the resulting recommendations for minor lexical adjustments were incorporated. A formal content validity index was not computed.

The questionnaire comprised three sections. The first section collected informed consent. The second section gathered sociodemographic and health information, including age, gender, place of residence, educational level, employment status, socioeconomic status, marital status, smoking status, self-reported body mass index (calculated from self-reported height and weight), personal history of chronic diseases, and family history of chronic diseases.

The third section assessed CVD knowledge, perceptions, and practices across multiple domains: general awareness of CVD (whether participants had heard of CVD and their sources of information), knowledge of CVD risk factors (using a closed-ended checklist of 11 items: aging, overweight/obesity, hypertension, diabetes, smoking, alcohol consumption, physical inactivity, family history of stroke, general stress, lipid disorders, and chronic kidney disease), awareness of CVD recurrence risk, knowledge of CVD warning signs (using a closed-ended checklist of 10 items: chest pain, shortness of breath, sweating, arm pain/numbness, loss of consciousness, headache, nausea/vomiting, dizziness, general fatigue/lethargy, and jaw/teeth pain), knowledge of preventive measures, knowledge of heart-healthy foods, perceived personal CVD risk, beliefs about CVD preventability, anticipated emergency response to a cardiovascular event, and cardiovascular health behaviors undertaken in the past year.

### 2.6. Outcome Measures and Scoring

The primary outcome was CVD knowledge, measured using a composite scoring approach consistent with the methodology of the parent instruments [25,26]. Knowledge scores were computed from two domains based on the closed-ended checklist responses. For knowledge of risk factors, each correctly identified risk factor from the 11-item checklist was assigned a score of 1, yielding a subscale score ranging from 0 to 11. For knowledge of warning signs, each correctly identified warning sign from the 10-item checklist was assigned a score of 1, yielding a subscale score ranging from 0 to 10. Participants who reported not knowing any risk factors or warning signs were assigned a score of 0 on the respective subscale, consistent with the “not knowledgeable” classification in the parent instruments. The two subscale scores were summed to produce a total CVD knowledge score ranging from 0 to 21.

Total knowledge scores were classified into four categories adapted from the cutoff criteria used in the parent studies: good knowledge (≥15 points), moderate knowledge (8–14 points), poor knowledge (1–7 points), and not knowledgeable (0 points). For binary logistic regression analysis, knowledge was dichotomized into adequate (good + moderate, score ≥ 8) versus inadequate (poor + not knowledgeable, score < 8). The ≥8 threshold was retained primarily for direct comparability with the parent instruments, in which the same cutoff distinguished knowledgeable from non-knowledgeable respondents. In addition, a score of ≥8 requires correct identification of at least 8 of the 21 listed risk factors and warning signs, distributed across both domains rather than concentrated in one, which was judged to represent a minimum practical level of awareness for a general-population screening instrument. This threshold should nonetheless be interpreted as a relative cutoff for between-group comparison rather than a clinically validated standard, and classification as “adequate” does not necessarily indicate knowledge sufficient for risk-factor modification or emergency symptom recognition during an actual cardiovascular event.

Internal consistency and reliability of the risk factor and warning sign subscales were evaluated using the Kuder-Richardson 20 (KR-20) coefficient, which is the appropriate form of Cronbach’s α for dichotomous items. Both subscales demonstrated strong internal consistency (KR-20: risk factors = 0.866; warning signs = 0.847; combined = 0.906), consistent with previous studies [25,26]. Internal consistency indicates that the items within each subscale measured a coherent underlying construct, but it does not by itself establish the validity of the combined 21-item score; because risk-factor and warning-sign knowledge represent conceptually distinct domains, the two subscale scores are also reported separately throughout the results.

### 2.7. Statistical Analysis

Data were analyzed using SPSS software (version 26.0; IBM Corp., Armonk, NY, USA). Categorical variables were summarized as frequencies and percentages, while continuous variables were reported as means ± standard deviations, with medians and interquartile ranges added for non-normally distributed variables. The Shapiro–Wilk test indicated that total CVD knowledge scores were not normally distributed (W = 0.931, *p* < 0.001); therefore, non-parametric tests were used for bivariate analysis. Mann–Whitney U tests (for two-group comparisons) and Kruskal–Wallis tests (for three or more groups) were used to examine associations between sociodemographic and health-related variables and total CVD knowledge scores. For each comparison, the test statistic (U with the corresponding z value for Mann–Whitney tests, and H with degrees of freedom for Kruskal–Wallis tests) is reported alongside medians and *p*-values. Variables with *p* ≤ 0.2 in the bivariate analysis were entered into a binary logistic regression model with knowledge adequacy (score ≥ 8 vs. <8) as the dependent variable. Logistic regression on the dichotomized outcome was selected because the total knowledge score was non-normally distributed, which violates the distributional assumptions of ordinary linear regression, and because the resulting adjusted odds ratios provide a clinically interpretable expression of the likelihood of adequate knowledge. The bivariate screening threshold of *p* ≤ 0.2, rather than the conventional *p* < 0.05, was used to retain candidate predictors with weaker bivariate but potentially meaningful adjusted associations; this data-driven approach is acknowledged as a limitation of the modelling strategy, as it may exclude a variable whose association emerges only after adjustment. Multicollinearity among predictor variables was assessed using variance inflation factors, with all values below 5. Model fit was assessed using the Hosmer–Lemeshow goodness-of-fit test and McFadden’s pseudo-R^2^. Results were reported as adjusted odds ratios (aOR) with 95% confidence intervals. Statistical significance was set at *p* < 0.05.

### 2.8. Ethical Considerations

Ethical approval was obtained from the Scientific Research Ethics Committee of Jazan University, Saudi Arabia (reference number: REC-45/07/971; approval date: 7 February 2024). Informed consent was obtained electronically from all participants prior to data collection. Participation was voluntary, and all data were anonymized and used exclusively for research purposes. The study was conducted in accordance with the Declaration of Helsinki.

## 3. Results

### 3.1. Sociodemographic and Health Characteristics

Table 1 summarizes the characteristics of the 382 participants. The majority were aged 18–29 years (61.5%), female (50.5%), rural residents (56.5%), university-educated (84.8%), and single (61.5%). Students comprised the largest employment group (43.7%). The mean BMI was 24.29 ± 5.63 kg/m^2^. Nearly one-third (29.6%) reported a personal chronic disease, and 71.2% had a family history of chronic disease.

### 3.2. CVD Awareness, Perceptions, and Practices

Most participants (89.5%) had heard of CVD, primarily through the internet/social media (66.1%) and family/friends (52.9%). Nearly half (49.5%) were uncertain about their personal CVD risk, though 80.6% believed CVD can be prevented. Regular exercise (84.3%) and maintaining an ideal weight (67.5%) were the most recognized preventive measures. In a cardiovascular emergency, 88.0% would take the person directly to the hospital. However, 19.1% reported doing nothing for cardiovascular health in the past year (Table 2).

### 3.3. Knowledge of Risk Factors and Warning Signs

As shown in Table 3, response patterns differed between the screening question and the closed checklist. In response to the screening question, 72.8% reported knowing at least one risk factor, and 63.4% reported knowing at least one warning sign. On the closed risk-factor checklist (11 items), the most-endorsed items were overweight/obesity (52.6%), hypertension (51.3%), and smoking (49.5%); chronic kidney disease (11.5%) was the least endorsed. On the closed warning-sign checklist (10 items), the most-endorsed items were chest pain (47.6%) and shortness of breath (46.1%); jaw/teeth pain (14.4%) was the least endorsed. The median total knowledge score was 7 (IQR 2–11) out of 21. Only 9.9% demonstrated good knowledge (≥15), while 53.7% had inadequate knowledge (<8).

### 3.4. Factors Associated with CVD Knowledge: Bivariate Analysis

Bivariate analysis using Mann–Whitney U and Kruskal–Wallis tests revealed significant associations between total CVD knowledge scores and age group (*p* < 0.001), education level (*p* = 0.014), employment (*p* < 0.001), marital status (*p* < 0.001), personal chronic disease (*p* < 0.001), and family chronic disease (*p* < 0.001) (Table 4).

### 3.5. Predictors of Adequate CVD Knowledge: Logistic Regression

Binary logistic regression identified two significant independent predictors of adequate CVD knowledge (Table 5). University education was associated with higher odds of adequate knowledge compared with high school or lower (aOR = 2.44, 95% CI 1.23–4.85, *p* = 0.011). Family history of chronic disease more than doubled the odds (aOR = 2.26, 95% CI 1.32–3.85, *p* = 0.003). As the study was cross-sectional, these associations indicate factors statistically associated with adequate knowledge and should not be interpreted as causal determinants.

## 4. Discussion

In this study, more than half of participants (53.7%) had inadequate CVD knowledge despite high general awareness (89.5%), establishing a marked awareness–knowledge gap as the central finding and the primary target for intervention. The good-knowledge proportion (9.9%) is well below the 61.5% reported in Syria [26] and below the 16.3% reported in rural Tanzania [25], and the median total knowledge score (7 of 21, IQR 2–11) is correspondingly low.

Within Saudi Arabia, our findings are among the lowest reported. A national Saudi study reported 55.8% strong CVD knowledge [16]; 60.7% demonstrated good knowledge in Najran [13]; a national coronary artery disease survey reported 70.3% with high knowledge scores [27]; and a western Saudi study reported only 18.5% knowledgeable about CVD risk factors [15]. Suboptimal knowledge has also been documented in the southern region [14], Jeddah [17,18], Kuwait [9], and Jordan [8]. The southern-region figure from this study (46.3% adequate; 9.9% good) sits in the lower portion of this range. Despite the low knowledge scores, general CVD awareness in this sample was high (89.5%), consistent with Syria (93%) [26] and other Saudi studies [12,16]. This persistent awareness–knowledge gap, in which individuals report having heard of CVD but cannot identify specific risk factors or symptoms, has been documented across sub-Saharan Africa in a systematic review of 20 studies [10] and indicates that passive exposure to health information does not translate into actionable knowledge. Because the design was cross-sectional, the present data cannot identify the causes of this gap, which may reflect the content or reach of health messaging, educational disparities, or characteristics of the measurement instrument itself.

Several factors may contribute to the lower position observed in the current study relative to other Saudi-region surveys. The rural majority in this sample (56.5%) is higher than reported in most national Saudi assessments, and rural residence is consistently associated with lower CVD knowledge in international studies [10]. Beyond sample composition, genuine regional differences in health-information ecosystems, including healthcare access, public-health messaging coverage, and educational attainment of the broader population, cannot be ruled out without comparable data from other Saudi regions.

University education and family history of chronic disease independently predicted adequate CVD knowledge in this study. Education was the strongest predictor (aOR = 2.44 for university vs. high school or lower, *p* = 0.011), consistent with findings from Tanzania [25], Syria [26], Kuwait [9], Jordan [8], and national Saudi studies [16,27]. Family history of chronic disease more than doubled the odds (aOR = 2.26, *p* = 0.003). Personal history of chronic disease showed a borderline association (aOR = 1.78, 95% CI 1.00–3.18, *p* = 0.052), consistent with the same perceived-susceptibility mechanism but not reaching conventional significance—possibly reflecting limited statistical power for this stratum (n = 113). These findings are consistent with the Health Belief Model, which posits that perceived susceptibility, heightened through personal or family experience with disease, motivates health information-seeking behavior [28]. The HBM constructs (perceived susceptibility, severity, benefits, barriers, self-efficacy) were not directly measured in this study, and the theoretical framing should be regarded as a post hoc interpretation rather than a confirmed mechanism. Bashatah et al. similarly reported that chronic disease presence was significantly associated with higher CVD knowledge among Saudi adults [16].

On the closed risk-factor checklist, overweight/obesity (52.6%), hypertension (51.3%), and smoking (49.5%) were the most-endorsed items; chronic kidney disease (11.5%) and lipid disorders (28.0%) were the least endorsed. These figures are substantially lower than those reported in Syria, where recognition rates exceeded 90% for smoking, obesity, and cholesterol [26], and lower than the national Saudi findings of 81.8% for hypertension and 78.7% for smoking [16]. The low recognition of diabetes as a CVD risk factor (34.8%) is particularly concerning given Saudi Arabia’s high diabetes prevalence [29]. Knowledge of cardiac-event warning signs (predominantly myocardial infarction) was even lower than knowledge of risk factors (mean 2.96/10 vs. 3.95/11), consistent with the pattern observed in Tanzania and Syria [25,26]. Chest pain was the most-endorsed item (47.6%) but recognition was markedly lower than the 87% reported in a national Saudi myocardial infarction awareness study [30], the 76.4% reported in Jeddah [17], and the 87.8% observed in Syria [26]. In Jordan, over 95% of caregivers of cardiology patients recognized chest pain, but only 53.5% identified jaw pain [31]—a pattern paralleled in this study, where jaw/teeth pain was the least-endorsed item (14.4%), consistent with 10.6% in Tanzania [25].

The poor recognition of cardiac-event warning signs has direct clinical consequences. A study of acute coronary syndrome survivors demonstrated that higher objective knowledge of CVD risk factors and symptoms was independently associated with shorter decision delay, with far fewer knowledgeable patients waiting more than one hour to seek help [32]. A systematic review of 57 studies on prehospital delay in acute coronary syndrome found that correct cardiac attribution of symptoms and perceived threat significantly shortened help-seeking time by 1.5 to 2 h [33]. Large registry data from 6544 patients with non-ST-segment elevation myocardial infarction demonstrated that prehospital delay of 24 h or more increased three-year all-cause mortality from 10.5% to 17% [34]. Although these prehospital-delay associations have not been replicated in Saudi populations specifically, the magnitude and consistency of the international evidence suggest the chain is likely to operate similarly in the present study setting. The low warning-sign knowledge observed in this sample, particularly the poor recognition of atypical presentations such as jaw pain, nausea, and fatigue, which differ from the classic chest-pain script and are recognized clinically as common in women, older adults, and patients with diabetes [35], may therefore be hypothesized to contribute to delayed emergency presentation; this possibility was not tested directly, as neither care-seeking behavior nor cardiovascular outcomes were measured in this study.

Despite a strong bivariate association (*p* < 0.001), age was not an independent predictor of knowledge after adjustment for covariates. This differs from Tanzania, where participants aged 45–54 had significantly higher knowledge [25], and Syria, where the same age group showed nearly fivefold higher odds [26]. In this study, the bivariate age effect was attenuated to non-significance after adjustment for marital status and chronic disease history, suggesting confounding by these covariates rather than mediation per se. Gender was not significantly associated with knowledge even at the bivariate level (*p* = 0.923) and was therefore not included in the regression model. This is consistent with findings from Jordan [8], though a Saudi national study reported that females exhibited greater awareness of coronary artery disease risk factors than males [27], and in the western region, older women and those with postgraduate education demonstrated the highest knowledge levels [36].

Several behavior patterns in this sample reinforce the prehospital delay concern raised above. Nearly half (49.5%) reported being unsure of their personal CVD risk, comparable to rural Tanzania (45.3%) [25]. Although 80.6% believed CVD is preventable, 19.1% reported no specific past-year cardiovascular health-related action, a belief–behavior disconnect also observed in national Saudi studies [12,16]. While 88.0% would take a person directly to the hospital during a cardiovascular emergency, a recent Al-Hasa study found that many Saudi adults delayed care-seeking due to cost and embarrassment [37], suggesting that correct knowledge of the appropriate response may not guarantee timely action; the small proportions endorsing traditional healers (0.5%), home treatment (0.8%), or waiting for recovery (0.3%) represent potentially fatal knowledge gaps. Together, these patterns of personal-risk uncertainty, belief–behavior disconnect, and incomplete emergency-response readiness provide further evidence consistent with the international chain linking suboptimal CVD knowledge to delayed presentation and adverse outcomes [32,33,34], strengthening the bridge from the observed knowledge gap to actionable intervention.

### 4.1. Limitations

Several limitations should be noted. The sample size, while exceeding the calculated minimum, remains modest for stratified subgroup analyses. The convenience-sampling approach and online distribution introduced selection bias toward digitally active respondents and may have under-represented less-digitally connected segments of the population, including older adults and those with lower educational attainment; self-report introduces response bias, including recall limitations and social-desirability effects. In particular, body mass index was derived from self-reported height and weight, and preventive behaviors were based on recall, so the high proportions reporting regular exercise and weight-management behaviors may overstate actual practice. The closed-ended checklist format may also have produced cueing effects, whereby respondents recognized listed items rather than recalling them spontaneously, potentially inflating measured knowledge relative to unprompted recall. Dichotomizing the knowledge score at the ≥8 threshold simplifies interpretation but reduces statistical information compared with the continuous score. Finally, the regression model explained only a modest share of the variance in knowledge (McFadden pseudo-R^2^ = 0.154; classification accuracy = 65.2%), indicating that much of the variation remained unexplained; unmeasured factors such as health literacy, healthcare access, prior exposure to health campaigns, and digital health engagement may account for additional variance and were not assessed. The cross-sectional design precludes causal inference.

### 4.2. Implications

These findings have several implications. First, the low knowledge despite high awareness indicates that current health communication strategies are insufficient, given that over 90% of Saudi adults use social media for health information [38]. Digital platforms represent a possible channel for delivering specific, actionable CVD education content rather than general awareness messages, although their effectiveness for improving CVD knowledge was not evaluated in this study and would require prospective testing. Second, interventions should target individuals with lower education (aOR = 2.44 for university vs. high school or lower) through community health workers, visual media, and simplified Arabic-language materials. Such education should prioritize the least-recognized items identified in this study: atypical warning signs (jaw or teeth pain, nausea or vomiting, and fatigue) and under-recognized risk factors (chronic kidney disease, diabetes, and lipid disorders). Third, healthcare providers should be activated as CVD education channels within routine primary care encounters, analogous to the health talk model used in reproductive health clinics [25]; family-history-cluster education (aOR = 2.26) using existing disease-history intake prompts may operationalize this. We acknowledge that the present sample under-represents the groups these interventions most need to reach (low-education 15.2%; aged ≥45 15.7%). These recommendations align with the Saudi Vision 2030 prioritization of noncommunicable disease prevention [4]. Future research should employ probability sampling, interviewer-administered questionnaires, and longitudinal designs to assess the effectiveness of targeted CVD education interventions in the Jazan region, Saudi Arabia.

## 5. Conclusions

This study found that more than half of the surveyed adults in the Jazan region, Saudi Arabia, had inadequate CVD knowledge despite high general awareness. Knowledge of warning signs was particularly poor, a gap that may be relevant to prehospital delay and adverse cardiovascular outcomes. Education level and family chronic disease history were the two significant independent predictors of adequate knowledge. These findings indicate substantial knowledge gaps among surveyed adults and support the development and evaluation of targeted health education programs that leverage digital platforms, activate healthcare providers as knowledge channels, and deliver specific, actionable information about CVD risk factors and warning signs. Whether such programs reduce the cardiovascular disease burden in the region remains to be established in future intervention studies.

## Figures and Tables

**Table 1:** Demographic and Clinical Characteristics of Participants (N = 382).

Variable	n (%)	Variable	n (%)
Sociodemographic Characteristics			
Age Group (29.78 ± 11.42 years)		Employment	
18–29	235 (61.5%)	Student	167 (43.7%)
30–44	87 (22.8%)	Full-time Employee	84 (22.0%)
≥45	60 (15.7%)	Currently Unemployed	83 (21.7%)
Gender		Part-time Employee	48 (12.6%)
Male	189 (49.5%)	Family Income Level	
Female	193 (50.5%)	High	28 (7.3%)
Place of Residence		Medium	321 (84.0%)
Urban	166 (43.5%)	Low	33 (8.6%)
Rural	216 (56.5%)	Marital Status	
Education Level		Single	235 (61.5%)
University	324 (84.8%)	Married	136 (35.6%)
High school or lower	58 (15.2%)	Divorced/Widowed	11 (2.9%)
Clinical and Health Characteristics			
BMI Category (24.29 ± 5.63 kg/m^2^)		Smoking Status	
Underweight	50 (13.1%)	Non-smoker	332 (86.9%)
Normal	185 (48.4%)	Current smoker	35 (9.2%)
Overweight	99 (25.9%)	Former smoker	15 (3.9%)
Obese	48 (12.6%)		
Chronic Disease Type—Self (multiple responses permitted) ^1^		Family History of Chronic Disease (multiple responses permitted) ^2^	
No	269 (70.4%)	No	110 (28.8%)
Yes	113 (29.6%)	Yes	272 (71.2%)
Diabetes	62 (16.2%)	Diabetes	222 (58.1%)
Hypertension	62 (16.2%)	Hypertension	198 (51.8%)
Obesity	48 (12.6%)	Obesity	56 (14.7%)
Lipid Disorders	46 (12.0%)	Lipid Disorders	59 (15.4%)
Thyroid Disorders	19 (5.0%)	Thyroid Disorders	43 (11.3%)
Chronic Lung Disease	14 (3.7%)	Chronic Lung Disease	24 (6.3%)
Asthma	2 (0.5%)	Chronic Kidney Disease	2 (0.5%)
Other ^1^	5 (1.3%)	Other ^2^	2 (0.5%)

^1^ Includes Heart Disease, H. Pylori, Focal Epilepsy, Sickle Cell Anemia, Hormonal Disorder, Cervical Spondylosis, PCOS. ^2^ Includes Colon and Stomach Disease, Heart Disease. Multiple responses were permitted for chronic-disease subcategories; percentages do not sum to total.

**Table 2:** CVD Awareness, Perceptions, and Practices (N = 382).

Variable	n (%)	Variable	n (%)
Ever heard of CVD		Perceived risk-reducing behaviors	
Yes	342 (89.5%)	Regular Exercise	322 (84.3%)
No	40 (10.5%)	Maintaining Ideal Weight	258 (67.5%)
Source of Information (n = 342) *		Avoiding Smoking and Alcohol	253 (66.2%)
Internet/Social Media	226 (66.1%)	Reducing Fatty Foods/Fast Food	251 (65.7%)
Family/Friends	181 (52.9%)	Regular Doctor Visits	217 (56.8%)
Ministry of Health	159 (46.5%)	Reducing Salt Intake	205 (53.7%)
Television	103 (30.1%)	Adhering to Medications	201 (52.6%)
Radio	59 (17.3%)	Do not know	26 (6.8%)
Know recurrence risk		Action in case of CVD event	
Yes	264 (69.1%)	Take to hospital directly	336 (88.0%)
No	118 (30.9%)	Do not know	30 (7.9%)
Self-perceived risk of CVD		Other responses	16 (4.2%)
Yes	111 (29.1%)	Past-year health actions	
No	82 (21.5%)	Weight management	231 (60.5%)
Do not Know	189 (49.5%)	Regular exercise	195 (51.0%)
Think CVD can be prevented		Reduced fatty food	150 (39.3%)
Yes	308 (80.6%)	Regular Medical Checkups	126 (33.0%)
No	15 (3.9%)	Reading About CVD	121 (31.7%)
Do not Know	59 (15.4%)	Reducing Salt in Diet	117 (30.6%)
Heart-healthy foods identified		Avoiding Smoking/Alcohol	114 (29.8%)
Leafy Vegetables	312 (81.7%)	Nothing	73 (19.1%)
Fruits	292 (76.4%)		
Wheat and Derivatives	78 (20.4%)		
Do not Know/Incorrect †	77 (20.2%)		

* Percentages based on respondents who had heard of CVD (n = 342); totals exceed 100% due to multiple responses. † Do not Know/Incorrect includes respondents who selected “Do not Know” or incorrect food items (salty foods, fast food, fatty foods).

**Table 3:** Knowledge of Risk Factors and Warning Signs, Scores, and Reliability (N = 382).

Risk Factors	n (%)	Warning Signs	n (%)
(A) Self-Reported Knowledge			
Yes	278 (72.8%)	Yes	242 (63.4%)
No	104 (27.2%)	No	140 (36.6%)
(B) Items Identified from Checklist			
Overweight/Obesity	201 (52.6%)	Chest Pain	182 (47.6%)
Hypertension	196 (51.3%)	Shortness of Breath	176 (46.1%)
Smoking	189 (49.5%)	Sweating	127 (33.2%)
Aging	168 (44.0%)	Arm Pain/Numbness	126 (33.0%)
Physical Inactivity	146 (38.2%)	Loss of Consciousness	107 (28.0%)
Diabetes	133 (34.8%)	Nausea/Vomiting	95 (24.9%)
Alcohol Consumption	113 (29.6%)	Headache	94 (24.6%)
Lipid Disorders	107 (28.0%)	Dizziness	89 (23.3%)
Family History of Stroke	107 (28.0%)	General Fatigue/Lethargy	81 (21.2%)
General Stress	105 (27.5%)	Jaw/Teeth Pain	55 (14.4%)
Chronic Kidney Disease	44 (11.5%)		
(C) Number of Items Identified			
None	104 (27.2%)	None	140 (36.6%)
1–4	112 (29.3%)	1–4	115 (30.1%)
5–11	166 (43.5%)	5–10	127 (33.2%)
Mean ± SD	3.95 ± 3.35	Mean ± SD	2.96 ± 2.89
(D) Total Knowledge Score (0–21) and Classification			
Median (IQR)	7 (2–11)	Mean ± SD	6.91 ± 5.64
Good knowledge (≥15)	38 (9.9%)	Adequate (≥8)	177 (46.3%)
Moderate knowledge (8–14)	139 (36.4%)		
Poor knowledge (1–7)	126 (33.0%)	Inadequate (<8)	205 (53.7%)
Not knowledgeable (0)	79 (20.7%)		

**Table 4:** Bivariate Analysis—Association Between Sociodemographic Variables and Total CVD Knowledge Score (N = 382).

Variable	Category	n	Median	Test Statistic	*p*-Value
Age Group	18–29	235	5	H(4) = 24.395	<0.001
	30–44	87	9		
	≥45	60	10		
Gender	Male	189	7	U = 18,134.5, z = 0.096	0.923
	Female	193	6		
Place of Residence	Urban	166	8	U = 16,089.5, z = 1.728	0.084
	Rural	216	5		
Education Level	University	324	7	U = 7506.5, z = 2.453	0.014
	High school or lower	58	4		
Employment	Student	167	5	H(3) = 17.036	<0.001
	Full-time Employee	84	8		
	Part-time Employee	48	10		
	Currently Unemployed	83	6		
Family Income	Low	33	4	H(2) = 4.154	0.125
	Medium	321	7		
	High	28	4		
Marital Status	Single	235	4	H(2) = 28.082	<0.001
	Married	136	10		
	Divorced/Widowed	11	4		
BMI Category	Underweight	50	4	H(3) = 4.630	0.201
	Normal	185	7		
	Overweight	99	8		
	Obese	48	6		
Chronic Disease (Self)	No	269	5	U = 10,781.0, z = 4.509	<0.001
	Yes	113	9		
Family Chronic Disease	No	110	3	U = 10,060.5, z = 5.041	<0.001
	Yes	272	8		
Smoking Status	Non-smoker	332	7	H(2) = 2.678	0.262
	Current smoker	35	5		
	Former smoker	15	6		

Note: Mann–Whitney U test (two-group comparisons). Kruskal–Wallis test (three or more groups).

**Table 5:** Binary Logistic Regression—Predictors of Adequate CVD Knowledge (N = 382).

Variable	aOR	95% CI	*p*-Value	Sig
Age 30–44 (ref: 18–29)	1.63	0.68–3.89	0.270	
Age ≥ 45 (ref: 18–29)	2.14	0.78–5.89	0.140	
University (ref: High school or lower)	2.44	1.23–4.85	0.011	*
Rural (ref: Urban)	0.69	0.43–1.11	0.128	
Full-time (ref: Student)	0.58	0.25–1.34	0.204	
Part-time (ref: Student)	1.34	0.43–4.19	0.615	
Unemployed (ref: Student)	0.80	0.41–1.59	0.529	
Low income (ref: Medium)	0.69	0.30–1.62	0.400	
High income (ref: Medium)	0.96	0.40–2.28	0.927	
Married (ref: Single/DW)	2.02	0.98–4.20	0.058	
Chronic disease—self (ref: No)	1.78	1.00–3.18	0.052	
Family chronic disease (ref: No)	2.26	1.32–3.85	0.003	**

Note: Dependent variable: adequate CVD knowledge (score ≥ 8, n = 177, 46.3%) versus inadequate (score < 8, n = 205, 53.7%). aOR = Adjusted Odds Ratio; CI = Confidence Interval. ** *p* < 0.01, * *p* < 0.05. Divorced/Widowed (n = 11) collapsed with Single as reference. Model: McFadden R^2^ = 0.154; Hosmer–Lemeshow χ^2^ = 10.21, *p* = 0.251; classification accuracy = 65.2%. All VIF < 5.

## Data Availability

The data presented in this study are available on reasonable request from the corresponding author. The data are not publicly available due to ethical and privacy restrictions (protection of participant confidentiality and in accordance with the approval granted by the Scientific Research Ethics Committee of Jazan University).
